# Contribution to the study of anthelmintic resistance of sheep gastro-intestinal strongyles on a sheep farm in the Guelma region, north-east Algeria

**DOI:** 10.2478/helm-2025-0014

**Published:** 2025-09-30

**Authors:** S. Djemai, N. Zeghilet, L. Boultif, O. Ayadi, Z.R. Djafar

**Affiliations:** 1 Laboratoire de Recherche de Pathologie Animale Développement des Elevages et Surveillance de la Chaine Alimentaire des Denrées Animales ou d’origine Animale (PADESCA Laboratory), Institute of Veterinary Sciences, University of Constantine 1, Algeria; 2 Institute of Veterinary Sciences, University of Constantine 1, Algeria; 3 Laboratoire de parasitologie, Ferhat Abbas University of Setif 1, Algeria

**Keywords:** Anthelmintics, gastrointestinal nematodes, resistance, sheep, Algeria

## Abstract

The prolonged use of anthelmintics is sometimes accompanied by a gradual decline in their effectiveness, refl ecting the development of resistant parasite strains. This study was conducted in the region of Guelma (northeastern Algeria) at the end of July and throughout August 2018 to investigate the sensitivity (or resistance) of sheep gastrointestinal nematodes to three anthelmintic products used in the Algerian fi eld. The sheepfold comprises 45 ewes of the Ouled-Djellal breed. The data showed that after treatment with anthelmintics, a signifi cant decrease in gastrointestinal nematode eggs per gram of feces (EPG) was observed in all groups. Resistance and sensitivity were observed in all groups studied. In group 1 (AL-Bendazol 2.5 %: albendazole), sensitivity Faecal Egg Count Reduction (FECR > 95 %) was noted in 2 out of 15 ewes (13 %), while resistance (FECR<95 %) was observed in 13 subjects (86.67 %). In the second group (Valbazen: albendazole), the numbers of cases in sensitivity and resistance were around 5 (33 %) and 10 (67 %), respectively. Group 3 (OXFENIL^®^ 2.265 %) revealed 2 (13 %) cases of sensitivity to oxfendazole and 13 resistance cases (86.67 %). No signifi cant difference was noted in the obtained results (sensitivity and resistance) between the three groups.

## Introduction

Gastrointestinal helminthiases are considered among the dominant parasitic diseases encountered in ruminant farms; they do not only affect the production performance, but can also cause clinical signs: anaemia, reproductive disorders, decline in the quality and quantity of milk, growth retardation, delayed puberty and mortality (Sheferaw *et al*., 2013; Saidi *et al*., 2020; Maestrini *et al*., 2021). They are generally attributable to nematodes and cestodes (*Nematodirus* spp., *Trichostrongylus* spp., *Teladorsagia circumcincta, Haemonchus contortus, Moniezia expansa*, etc.) (Martínez-Valladares *et al*., 2015). Controlling these intestinal parasitic diseases is a crucial factor in optimizing animal production. Preventive anthelmintic chemotherapy remains the most widely used means of preventing these pathologies. The three classes of anthelmintics most commonly used in ruminants are benzimidazoles, macrocyclic lactones, and cholinergic agonists (in particular, levamisole) (Benguesmia *et al*., 2020; Potârniche *et al*., 2021). The excessive use of these anthelmintics can induce chemoresistance, especially since an increase in the resistance of gastrointestinal nematodes (to anthelmintics) has been reported in many countries in sheep and goats (Martínez-Valladares *et al*., 2015; Maestrini *et al*., 2021). In Algeria, few studies have been carried out on the resistance to anthelmintics in small ruminants; we can note, as an example, the study of Bentounsi *et al*. (2006), which demonstrated resistance of gastrointestinal nematodes in sheep to two benzimidazoles (albendazole, fenbendazole).

This study, conducted on ewes belonging to a farm located in the region of Guelma (north-eastern Algeria), aims to assess the resistance of gastrointestinal nematodes against two generics of albendazole and one of oxfendazole and this, by performing the Faecal Egg Count Reduction Test (FECRT): in-vivo test recommended by the World Association for Advance in Veterinary Parasitology (WAAVP) for the evaluation of the anthelmintic resistance in gastrointestinal strongyles in ruminants, horses and pigs (Coles *et al*.,1992).

## Materials and Methods

### Study area and experiment design

The current study was conducted on a sheepfold in the neighborhood of Djeballa Khmissi, Wilaya of Guelma, northeastern Algeria, in late July and early August of 2018. The climate of this locality is semi-arid with a mean annual rainfall ranging between 450 and 600 mm. The sheepfold comprises 90 ewes of the Ouled-Djellal breed, aged between 2 and 5 years (mean age = 4.16 years), under semi-extensive breeding conditions. Animals were fed during late autumn, winter, and early spring with a concentrate consisting of corn, wheat bran, and barley. From summer until the beginning of autumn, the animals grazed on pastures. All animals are dewormed every 4 months with albendazole and ivermectin.

To assess the presence of nematode resistance to deworming, a total number of 45 ewes not dewormed for a little over 3 months were randomly divided into three batches (15 animals/group): animals of groups 1, 2 and 3 are dewormed respectively by albendazole (Al-Bendazol 2.5 % / Algérian Animal Health Product “AAHP”), albendazole (Valbazen^®^- Sheep and Goats / ZOETIS) and oxfendazole (OXFENIL^®^ 2.265 % / VIRBAC).

### Experimental protocol

On the first day, the animals in each of the three groups are given the appropriate anthelmintic (group 1 receives Al-Bendazol 2.5 % AAHP, group 2 receives Valbazen^®^, and group 3 receives OXFENIL^®^ 2.265 %).

Faecal samples were collected in identified bags and sent to the laboratory on day 1 (just before treatment) and from day 10 to 14 after treatment. The modified McMaster method (Taylor *et al*., 1995) was performed to estimate the number of eggs per gram of faeces (EPG).

The percentage of egg reduction was estimated by the Faecal Egg Count Reduction Test (FECRT) as follows (Eysker *et al*., 2000):

$$\text{FECR}=\frac{\left(\text{EPG}1-\text{EPG}2\right)}{\text{EPG}1}\times 100$$

FECR (Faecal Egg Count Reduction) represents the reduction in the number of eggs of gastrointestinal nematodes after deworming. EPG_1_ is the Number of eggs per gram of faeces before treatment, calculated by the modified McMaster method on the sample taken on day 0. EPG_2_ represents the number of eggs per gram of feces after treatment, calculated by averaging the EPGs between days 10 and 14 post-treatment. The parasite was considered resistant if the FECR was lower than 95; otherwise, it was considered sensitive.

### Statistical analysis

The data were analyzed using the chi-square test in IBM SPSS Statistics (version 24) at a 5 % threshold.

## Ethical Approval and/or Informed Consent

The study was reviewed and approved by the local animal experimentation ethics committee at the University of Constantine-1.

Experimental and rearing conditions in this study comply with the animal welfare standards outlined by Oliver and Rossenrode (2017).

## Results

The EPG_1_ values in the three groups (before treatment) were very similar, ranging from 150 to 1,600 EPG. After treatment with anthelmintics, we observed a decrease in the number of gastrointestinal nematode eggs in the groups (Table 1). In group 1, the mean value of EPG_1_ was around 803; after treatment with “AL-Bendazol 2.5 %,” the number of nematode eggs per gram of feces decreased, reaching an average value of about 97 (EPG_2_). The average EPG_1_ value obtained in group 2 was around 450; deworming with Valbazen^®^ reduced the excreted (in the faeces) number of digestive nematode eggs to an average value of around 50 (EPG_2_). The average OPG_1_ calculated in group 3 was approximately 353; the number of nematode eggs excreted in the feces after treatment with Oxfenil decreased to an average value of approximately 87 (EPG_2_) (Table 1). Resistance and sensitivity were observed in each of the three batches. In batch 1, resistance to “AL-Bendazol 2.5 %” was observed in 13 cases out of 15 ewes (86.67 %). In the second group, the number of cases demonstrating resistance to “Valbazen” was 10 out of 15 ewes (67 %). Group 3 reveals 13/15 (86.67 %) cases of resistance to “oxfendazole” (Table 2). The number of cases showing sensitivity to the used products was: 2/15 (13.33 %), 5/15 (33.33 %), and 2/15 (13.33 %), respectively, in groups 1, 2, and 3 (Table 2).

**Table 1.** The Faecal egg count reduction and the number of eggs per gram of faeces (before and after treatment).

Group*		Group 1	Group 2	Group3
**EPG_1_**	**Average ± SD**	803.3 ± 329.8	450 ± 275.2	353.3 ± 246.7
	**Range (Mini – Max)**	150 – 1600	150 – 1050	150 – 1150
**EPG_2_**	**Average ± SD**	96.7 ± 66.7	50.0 ± 50.0	86.7 ± 71.2
	**Range (Mini – Max)**	0 – 250	0 – 150	0 – 250
**FECR_1_(%) S**	**Average ± SD**	100 ± 0	100 ± 0	100 ± 0
	**Range (Mini – Max)**	100 – 100	100 – 100	100 – 100
	**Number of cases**	2	5	2
**FECR_2_ (%) R**	**Average ± SD**	85.7 ± 7.1	81.9 ± 9.1	69.5 ± 19.0
	**Range (Mini – Max)**	70.6 – 94.1	66.7 – 94.1	16.7 – 91.3
	**Number of cases**	13	10	13

* 15 ewes in each group, EPG_1_: Number of Egg Per Gram of Faeces before anthelminthic treatment, EPG_2_: Number of Egg Per Gram of faeces after anthelmintic treatment, FECR_1_: Faecal Egg Count Reduction ⩾95%, FECR_2_: Average Faecal Egg Count Reduction < 95%, R: Resistant, S: Sensible, SD: Standard deviation

**Table 2.** Resistance and sensitivity observed in the three tested groups.

Group*	Sensitivity Number of cases (%)		Resistance Number of cases (%)
**Group 1 (AL-Bendazol 2.5%)**	2 (13.33%)		13 (86.67%)
**Group 2 (Valbazen)**	5 (33.33%)		10 (66,67%)
**Group 3 (Oxfenil 2.265%)**	2 (13.33%)		13 (86.67%)
***p-*value**			
**(1 vs 2)**	0.344	0.874	0.591
**(1 vs 3)**	0.454	0.439	0.551
**(2 vs 3)**	0.069	0.507	0.283

The statistical comparison of the three products used did not reveal any significant difference in terms of deworming efficacy (Table 2).

## Discussion

Resistance of gastrointestinal nematodes to anthelmintics, particularly benzimidazoles, has been widely observed worldwide (Höglund *et al*., 2015). In our study, the resistance to albendazole (through the two specialties used) and to oxfendazole is observed, thus indicating a reduction in the effectiveness of these treatments in Egypt, Aboelhadid *et al*. (2021) found resistance to albendazole with an FECR of around 86.68 %. Bentounsi *et al*. (2007) also observed resistance of digestive strongyles to benzimidazoles in five pilot sheep farms located in eastern Algeria. The continuous and repeated use of albendazole in the farm has been studied for about 26 years (since the establishment of this farm). The relatively high frequency of treatment (three treatments per year), may have allowed the development of resistance to benzimidazole molecules including those used in this study (albendazole and oxfendazole), knowing that many authors attest that the intensive use of the same anthelmintic leads to the emergence of populations of nematodes resistant to the used molecule (Besier, 2007; Falzon *et al*., 2014). The problems in dosage errors, namely overdose and underdose, also represent an essential factor in the development of resistance, especially since in Algerian farms (cattle, sheep and goats) the evaluation of the dose of an anthelmintic is generally made by approximate visual weight estimate of the animal, which can induce an underdose or an overdose, allowing the selection of resistant helminths populations (Smith *et al*., 1999; Besier, 2007; Falzon *et al*., 2014).

The semi-intensive system of the studied farm ensures a continuous parasitic infestation of animals, favouring the development of eggs and the contamination of pastures for weeks, which increases the pressure of parasitic infestation on sheep. This can, in turn, increase the risks of selecting resistant populations (Nascimento *et al*., 2021).

For some authors, populations of resistant nematodes may be introduced into the land of a given country by herds of animals imported from another country. In Sweden, Höglund *et al*. (2015) observed the emergence of *Haemonchus contortus* populations resistant to ivermectin in certain sheep farms. The authors partly attributed this resistance to the introduction of resistant parasites via imported animals from Europe, where resistance to avermectins has been reported.

This study enabled us to observe the presence of resistance in digestive strongyles to anthelmintics, thereby corroborating previous observations by some authors, notably Bentounsi *et al*. (2006) and Boulkaboul *et al*. (2010), who had already reported the existence of resistance to anthelmintics in Algeria.

Albendazole is considered the most widely used anthelmintic in the Algerian field; this requires farmers to carry out correct management of their herds, based on respecting doses, alternating between families of anthelmintics, and not introducing resistant parasites into the herd (e.g., quarantine and association of anthelmintics).

## Conclusion

This survey demonstrated the presence of resistance to benzimidazoles in gastrointestinal strongyles of sheep. Further studies on the effectiveness of other molecules from different families of anthelmintics should be pursued in the Algerian context. Specific analyses are necessary to determine nematode species who might exhibit chemoresistance.
